# Associations of physical activity types and intensity with cardiovascular diseases by age and gender among 18,730 Chinese adults

**DOI:** 10.1038/s41598-023-41768-0

**Published:** 2023-09-05

**Authors:** Beilei Lin, Zhenxiang Zhang, Weihong Zhang, Chunhui Zhang, Lihong Xue, Baoxia An, Kaijuan Wang

**Affiliations:** 1 Nursing and Health School of Zhengzhou University, Zhengzhou, China; 2 Huaxian People Hospital of Henan Province, Anyang, China; 3 Public Health School of Zhengzhou University, Zhengzhou, China

**Keywords:** Cardiovascular diseases, Risk factors

## Abstract

The associations of physical activity (PA) intensity and types with CVD (cardiovascular diseases) in different population are inconsistent and remains not very clear. A total of 7854 males and 10,876 females over 15 years were selected by multistage random sampling methods. In males, moderate-intensity physical activity (MPA) had no effect, while vigorous-intensity physical activity (VPA) played a significant protective role (OR = 1.319 and 0.615). However, in females, both MPA and VPA had significant protective effects (OR = 0.593 and 0.537). VPA presented as a significant protective factor in stroke patients and combined CVDs for males in all age groups; however, in females, the results suggested that, for those aged over 60–74 years, MPA was a protective factor. Furthermore, for the middle-aged or younger participants, the area under the curves (AUCs) of work, housework, and leisure activity were generally higher than that of other types, while for patients aged over 60 years, the AUCs of sedentary time and sleep activity seemed much higher. VPA had a better protective effect for preventing cardiovascular events, while the young and female population could benefit from MPA as well. Regardless of the types of PA, leisure activities were strongly recommended, and young people were much more likely to benefit from exercise than older people.

## Introduction

The burden of cardiovascular disease (CVD) remains a global health issue^1^. According to the World Health Organization (WHO)’s report, CVDs take the lives of 17.7 million people every year and are the leading cause of global death in both developed and developing countries^2^. As the American Heart Association (AHA) reported in 2023, the prevalence of CVD in adults aged ≥ 20 years old is 48.6% overall^3^. Additionally, China has encountered a heavy disease burden caused by CVDs because of the considerable economic, social, and environmental change in the past 40 years^4^, CVDs are the number one reason for death in both urban (41.06%) and rural (38.72%) areas^5^, and most of these are caused by stroke or myocardial infarction (MI). Furthermore, more young adults aged under 65 years suffered from stroke^6^, and a 24-year follow-up study by Ning suggested that the incidence of stroke in the younger population increased greatly, while in the older population, the epidemic trend remained steady or even slightly improved, which was in contrast with the pattern observed in some developed countries^7^. More seriously, the incidence of acute myocardial infarction (AMI) among the younger population is even higher than that in the middle-aged population in China^8^, and with the long-term effect of ambient air pollution, the incidence seems to be increasing each year^9^.

Physical inactivity (PA) is a leading risk factor for CVD and premature death worldwide^10^. Meanwhile, many studies^4, 5, 11–13^ have confirmed the importance of PA for cardiovascular health; keeping active can not only prevent the occurrence of CVDs but can also reduce mortality^14^. Previous studies have shown that lack of PA was an independent CVD risk factor, and various studies have shown that regular exercise could be a protective factor for patients with CVDs^15–19^; patients who are less physically active may have higher red blood cell aggregation, which could exacerbate the risk of stroke or stenosis^20^. In addition, in China, people tend to sit longer and perform less exercise, especially with the broader use of mobile equipment such as phones and iPads. Physical inactivity could lead to obesity and high levels of blood lipids, cholesterol, and even inflammatory markers^21, 22^, all of which have exacerbated the risk of CVDs^7, 12, 23^.

According to WHO, PA is a general term involving leisure time exercise, occupational physical activity, housework-related activity, or commuting^24, 25^. Although current studies explore the relationship of both leisure exercise and occupational activity with stroke and MI, it is still difficult to reach a consensus. As some studies have confirmed that vigorous-intensity leisure time exercise could reduce the risk of CVDs^26–28^, while other studies have shown that occupational PA did not affect cardiovascular health^17, 29^, and may increase the risk^16, 30^. However, according to a study by Zhao et al.^23^ sports-related activity with high frequency and long duration could prevent AMI among patients from Northern China. Another interesting finding of Johansson was that the association between PA and systolic blood pressure was inconsistent at leisure or work^31^. Despite the mixed findings, these studies only focused on the types or intensity of PA, and up to date, no study has examined the possible effects of age and gender on the associations. Meanwhile, which kinds and intensities of PA among different ethnic populations would be the most beneficial could provide more evidence as well. Thus, using the data from a cohort of 18,730 Chinese adults, the study aimed to explore the associations between PA and MI and stroke, and then to assess the associations between PA types, intensity, and CVDs stratified by age and gender.

## Methods

### Study design and participants

This cross-sectional epidemiological investigation was conducted in Henan Province. The methods and design of this study have been reported in detail by previous studies^32–34^. A multistage random sampling method was used to select a representative sample of 19,000 individuals from the permanent residents. First, four cities in urban areas and six counties in rural areas were selected using probability proportional to size (PPS) methods; second, two districts or towns were selected from each city or county by using simple random sampling (SRS); third, three communities or small villages were selected using SRS; and finally, 19,000 individuals aged > 15 years were chosen within all sample sites. Participants signed the written informed consent form before the face-to-face questionnaire interviews or physical measurements. The survey protocol was approved by the Ethical Committee of the Chinese Ministry of Science and Technology.

### Data collection methods

We trained postgraduates and undergraduates to conduct the investigation. The questionnaires included demographic data (such as gender, age, residential area, ethnicity, marital status, educational level, occupation, etc.), smoking and alcohol drinking status, self-report diseases, or family history. A physical examination was carried out to collect height, weight, waist circumference (WC), basal metabolism (BM), body mass index (BMI), percentage of body fat (PBF), visceral fat index (VFI), heart rate (HR), systolic blood pressure (SBP) and diastolic blood pressure (DBP). Height was measured to the nearest 0.5 cm without shoes using a standard stadiometer by trained observers. Weight (0.1 kg precision) was measured without heavy clothing and shoes with a weight-determined device (V-body HBF-371, OMRON, Kyoto, Japan). Waist circumference was measured to the nearest 0.5 cm midway between the lowest rib and the superior border of the iliac crest with flexible anthropometric tape. BM, PBF, and VFI were measured using an OMRON body fat and weight measurement device (V-body HBF-371, OMORON, Kyoto, Japan). The blood pressure was measured with both an OMRON device (OMRON, Kyoto, Japan) and a mercury sphygmomanometer (Yu-tu, Shanghai Medical Instruments Co., Ltd., Shanghai, China) three times on the right arm supported at heart level after the participant was sitting at rest for 5 min, with 30 s between each measurement.

The long-form International Physical Activity Questionnaire (IPAQ) was used to evaluate an individual’s moderate to vigorous physical activity or amount of sedentary behavior throughout the last seven-day week^35^. IPAQ can provide researchers with an estimate of PA and sedentary behavior for adults aged 15–69 years, and is better implemented in larger surveillance studies across a range of socio-economic settings^36^. Details about the intensity, duration (min/day), and frequency (days/week) of PA were undertaken within three domains (occupational, agricultural, and housework activity, transportation activity, and leisure time activity). Sedentary behavior and sleeping time per day were also recorded at the same time. The domain-specific PA intensity was quantified in the form of metabolic equivalents of tasks (METs) and then categorized into light, moderate, and vigorous groups based on the Chinese version of the guidelines^37^. The PA energy expenditure (PAEE) of each domain was calculated and summed for the total PAEE. The proportions of the PAEE in the work, transportation, and leisure-time domains and the total PAEE were calculated^38^.

### Statistical analysis

A total of 18,730 participants were included in the current analysis after excluding those who had incomplete physical examination or PA information. Continuous data are expressed as the mean $\pm$ standard deviation (M $\pm$ SD); discrete data are shown as frequencies or percentages; the t-test or Wilcoxon test and the chi-square test were used to analyze the continuous and discrete data, respectively. The anthropometric and bioelectrical indices were categorized into higher- and lower-level groups according to the age- and gender-specific optimal cutoff values proposed in the subsequent receiver operating characteristic (ROC) analyses. Crude odds ratios (ORs) were obtained from a binary logistic regression model and compared using the Breslow-Day test. Following the multicollinearity assessment among all the variables, multivariate logistic regression was performed to produce ORs by gender and age groups adjusting for age, residential area, education level, smoking status, drinking status, sedentary behavior, and family history of hypertension, stroke, and coronary heart disease. ROC analyses for men and women were employed to determine optimal cutoff values for each type of PA in relation to MI and stroke. Furthermore, we calculated the AUCs and 95% confidence intervals (CIs) for work activity, transportation activity, leisure-time PA, sedentary time, and sleeping time in discriminating MI and stroke, and the statistical significance was tested among them with the method of Delong et al. using MedCalc Version 10.1.6.0 (MedCalc Software, Ostend, Belgium)^32^. We calculated the PAR of multiple risk factors using the equation recommended by Yu from Fudan University^39^. All data were recorded and checked using EpiData version 3.1. The statistical analysis was conducted with SPSS version 21.0. All *P* values were two-sided with a significant level of 0.05.

## Results

### Basic characteristics of the participants

A total of 18,730 subjects aged 15 years and older were included in this study. This study included 7854 males (92.12% with non-CVDs, 1.46% with MI, 5.97% with stroke, and 0.44% with both MI and stroke (MS)) and 10,867 females (94.20% with non-CVDs, 1.11% with MI, 4.41% with stroke, 0.28% with both MI and stroke). Tables 1 and 2 show the demographic, disease-related, and metabolic characteristics of the study population. The average age of the male subjects was 51.34 years old, and the average age of the females was 52.74 years old; the average age of male stroke patients was 64.97 and female stroke patients was 64.70 years old. Age tended to be increased in the MI and stroke groups in both males and females (*P* < 0.001). Comparing the non-CVD group with the MI, stroke, or MS groups, the participants’ educational level, marital status, working status, drinking, hypertension, family history, body examination, and PA were significantly different both in males and females, and the grouped differed by smoking status only in the males (Tables 1 and 2).

**Table 1:** Demographic characteristics of male participants with or without cardiovascular diseases (N = 7854, n (%)).

Characteristics/Group	Non-CVDs (n1 = 7235)	MI (n2 = 115)	Stroke (n3 = 469)	MS (n4 = 35)	*OR*^*$*^	*95%CI*^*$*^	*χ*^*2*^*/F*	*P*
Age (years)	50.18 ± 17.13	63.57 ± 10.79	64.97 ± 9.57	67.46 ± 8.92	–	–	147.681	< 0.001
< 45	2677 (37.0)	4 (3.5)*	12 (2.6)*	1 (2.9)*	–	–	446.530	< 0.001
45–59	2054 (28.4)	37 (32.2)	106 (22.6)*	4 (11.4)	9.152	5.451–15.364		
60–74	1995 (27.6)	51 (44.3)*	276 (58.8)*^▲^	23 (65.7)*^▲^	24.786	14.981–41.010		
75–	509 (7.0)	23 (20.0)*	75 (16.0)*	7 (20.0)*	33.169	19.352–56.853		
Education level							136.948	< 0.001
~Illiterate	775 (10.7)	12 (10.3)	85 (18.1)*	3 (8.6)				
Primary school	1378 (19.0)	30 (26.1)	152 (32.4)*	12 (34.3)	1.065	0.687–1.653		
Middle school	2553 (35.3)	41 (35.7)	157 (33.5)	12 (34.3)	1.117	0.730–1.709		
High school	1322 (18.2)	24 (20.9)	62 (13.2)*	5 (14.3)	0.984	0. 605–1.602		
College–	1207 (16.6)	8 (7.0)	13 (2.8)*	3 (8.6)	0.531	0.263–1.075		
Marital status							82.674	< 0.001
Unmarried	984 (13.6)	2 (1.7)*	7 (1.5)*	0 (0.0)*	–	–		
Married	5807 (80.3)	104 (90.4)*	419 (89.3)*	30 (85.7)*	2.680	1.357–5.290		
Currently single	444 (6.1)	9 (7.9)	43 (9.2)	5 (14.3)	1.644	0.782–3.455		
Working status								
Employed	4069 (56.2)	36 (31.3)*	108 (23.0)*	4 (11.4)*			434.728	< 0.001
Retired	499 (6.9)	24 (20.9)*	78 (16.6)*^▲^	14 (40.0)*	3.126	2.153–4.540		
Students	551 (7.6)	0 (0.0)*	0 (0.0)*	0 (0.0)*				
Underemployed	2116 (29.2)	55 (47.8)*	283 (60.3)*	17 (48.6)	2.103	1.541–2.869		
Smoking							50.382	< 0.001
Never	3836 (53.0)	75 (65.3)*	317 (67.6)*	25 (71.4)*				
Occasionally	504 (7.0)	2 (1.7)	20 (4.3)*	1 (2.9)	0.697	0.532–0.914		
Always	2895 (40.0)	38 (33.0)	132 (28.1)	9 (25.7)	0.669	0.374–1.196		
Drinking							37.392	< 0.001
No	2861 (39.5)	63 (54.8)*	243 (51.8)*	17 (48.6)*				
Yes					0.603	0.512–0.711		
Daily	917 (12.7)	12 (10.4)	79 (16.8)*	7 (20.0)				
Weekly	1191 (16.5)	20 (17.4)	71 (15.1)	5 (14.3)	0.747	0.557–1.001		
Monthly	1171 (16.2)	11 (9.6)	42 (9.0)*	2 (5.7)	0.435	0.309–0.612		
Occasionally	1095 (15.1)	9 (7.8)	35 (7.5)*	4 (11.6)	0.406	0.284–0.579		
Hypertension							638.587	< 0.001
No	5987 (82.8)	80 (69.6)*	167 (35.6)*^▲^	15 (42.9)*^▲^				
Yes	1248 (17.2)	35 (30.4)*	302 (64.4)*^▲^	20 (57.1)*^▲^	5.262	4.016–6.895		
Family history							37.970	< 0.001
No	4432 (61.3)	53 (46.1)*	237 (50.5)*	15 (42.9)	–	–		
Yes	2701 (37.3)	58 (50.4)*	225 (48.0)*	19 (54.3)	1.941	1.629–2.312		
Unclear	102 (1.4)	4 (3.5)	7 (1.5)	1 (2.9)	1.676	0.884–3.175		
Body examination								
BMI	24.72 ± 3.63	25.37 ± 3.26	25.41 ± 3.52	25.92 ± 3.13	1.048	1.000–1.098	7.574	< 0.001
WC (cm)	87.68 ± 20.47	90.23 ± 9.25	90.76 ± 9.52	90.85 ± 8.59	1.001	0.994–1.008	4.350	0.005
BM	1574.38 ± 200.92	1552.52 ± 188.70	1521.71 ± 171.28	1519.17 ± 164.70	0.997	0.996–0.998	11.407	< 0.001
VFI	23.99 ± 13.52	27.94 ± 5.17	27.65 ± 5.44	28.39 ± 4.58	1.011	0.995–1.027	15.673	< 0.001
PBI (%)	11.06 ± 12.73	12.88 ± 5.11	13.25 ± 5.25	14.13 ± 5.61	1.014	0.993–1.035	6.024	< 0.001
Physical activity							71.289	< 0.001
Light	1018 (14.0)	19 (16.5)	81 (17.3)	5 (14.3)				
Moderate	1647 (22.8)	41 (35.7)*	170 (36.2)*	13 (37.1)	1.319	1.032–1.684		
Vigorous	4570 (63.2)	55 (47.8)*	218 (46.5)*	17 (48.6)	0.615	0.615–0.777		

BMI = body mass index; WC = waist circumstance; BM = basic metabolism; VFI = visceral fact index; PBF = percentage body fat.

*Compared with Non-CVD group, *P* < 0.05; ^▲^Compared with MI group, *P* < 0.05; ^*$*^CVD compared with Non-CVD group.

CVD = MI + stroke + MS, the total number is 619.

**Table 2:** Demographic characteristics of female participants with or without cardiovascular diseases (N = 10,876, n (%)).

Characteristics/Group	Non-CVDs (n1 = 10,245)	MI (n2 = 121)	Stroke (n3 = 480)	M–S (n4 = 30)	*OR*^*$*^	*95%CI*^*$*^	*χ*^*2*^*/F*	*P*
Age (year)	51.99 ± 16.62	65.94 ± 9.23	64.70 ± 8.34	66.33 ± 7.27	–	–	127.500	< 0.001
< 45	3027 (29.5)	1 (0.8)	5 (1.0)	2 (6.7)	–	–		
45–59	3337 (32.6)	25 (20.6)	113 (23.5)	24 (80.0)	21.166	9.355–47.989		
60–74	3171 (31.0)	74 (61.2)	312 (65.0)	4 (13.3)	65.230	29.090–146.269		
75–	710 (6.9)	21 (17.4)	50 (10.5)	0 (0.0)	53.292	23.109–122.896		
Education level							224.422	< 0.001
Illiterate	2867 (28.0)	46 (38.0)	229 (47.7)*	17 (56.7)*				
Primary school	2223 (21.7)	40 (33.1)*	155 (32.3)*	4 (13.3)*	0.879	0.728–1.061		
Middle school	2652 (25.9)	25 (20.7)	75 (15.6)*	7 (23.3)	0.396	0.315–0.497		
High school	1162 (11.3)	8 (6.6)	20 (4.2)*	2 (6.7)	0.253	0.173–0.371		
College–	1341 (13.1)	2 (1.7)*	1 (0.2)*	0 (0.0)*	0.029	0.009–0.090		
Marital status							112.661	< 0.001
Unmarried	1199 (11.7)	1 (0.8)*	4 (0.8)*	0 (0.0)				
Married	7761 (75.8)	90 (74.4)	372 (77.5)	21 (70.0)	2.331	0.939–5.791		
Currently single	1285 (12.5)	30 (24.8)*	104 (21.7)*	9 (30.0)*	1.742	0.678–4.477		
Working status							207.866	< 0.001
Employed	3598 (35.1)	13 (10.7)*	105 (21.9)*	7 (23.3)*				
Retired	605 (5.9)	23 (19.0)*	19 (4.0)	4 (13.3)	2.189	1.544–3.102		
Students	959 (9.4)	0 (0.0)*	0 (0.0)*	0 (0.0)*				
Underemployed	5083 (49.6)	85 (70.3)*	356 (74.2)*	19 (63.4)	2.605	2.128–3.189		
Smoking							5.660	0.462
Never	10,121 (98.8)	119 (98.4)	472 (98.3)	30 (100.00)				
Everyday	96 (0.9)	1 (0.8)	8 (1.7)	0 (0.0)	2.880	1.734–4.784		
Not everyday	28 (0.3)	1 (0.8)	0 (0.0)	0 (0.0)	0.582	0.194–3.332		
Drinking							29.131	< 0.001
No	9125 (89.1)	115 (95.0)*	462 (96.3)*	27 (90.0)*				
Yes					0.364	0.246–0.538		
Daily	92 (0.9)	2 (1.7)	8 (1.7)*	2 (6.7)*				
Weekly	108 (1.1)	1 (1.3)	2 (0.4)	1 (3.3)	0.284	0.089–0.911		
Monthly	169 (1.6)	1 (0.7)	3 (0.6)	0 (0.0)	0.136	0.037–0.495		
Occasionally	751 (17.5)	2 (1.3)	5 (1.0)*	0 (0.0)*	0.082	0.033–0.205		
Hypertension							566.448	< 0.001
No	7902 (77.1)	52 (43.0)*	146 (30.4)*^▲^	13 (43.3)				
Yes	2343 (22.9)	69 (57.0)*	334 (69.6)*^▲^	17 (56.7)	5.961	5.035–7.058		
Family history							36.231	< 0.001
No	4260 (41.6)	62 (51.2)*	224 (46.7)*	11 (36.7)*				
Yes	5810 (56.7)	53 (43.8)*	252 (52.5)*	18 (60.0)*	1.483	1.261–1.745		
Unclear	175 (1.7)	6 (5.0)*	4 (0.8)	1 (3.3)	1.230	0.661–2.286		
Body examination								
BMI	25.09 ± 5.86	26.03 ± 4.03	27.38 ± 21.17	26.28 ± 4.41	1.077	1.035–1.121	16.003	< 0.001
WC (cm)	84.41 ± 16.91	88.61 ± 10.36	89.75 ± 9.95	89.87 ± 11.36	1.000	0.995–1.004	19.023	< 0.001
BM	1264.88 + 168.48	1244.19 ± 149.39	1290.18 ± 431.07	1236.73 ± 175.42	1.000	0.999–1.001	3.552	0.014
VFI	33.63 ± 14.82	36.62 ± 4.82	38.41 ± 44.19	37.29 ± 5.35	0.980	0.955–1.005	13.298	< 0.001
PBI (%)	8.53 ± 20.14	10.15 ± 4.59	12.36 ± 45.38	13.47 ± 15.80	0.986	0.956–1.016	5.399	0.001
Physical activity							28.749	< 0.001
Light	711 (6.9)	16 (14.6)*	53 (11.0)*	6 (20.0)*				
Moderate	2733 (26.7)	34 (25.2)	133 (27.7)	4 (13.3)	0.593	0.447–0.788		
Vigorous	6801 (66.4)	71 (60.3)	294 (61.3)	20 (66.7)	0.537	0.414–0.695		

BMI = body mass index; WC = waist circumstance; BM = basic metabolism; VFI = visceral fact index; PBF = percentage body fat.

*Compared with Non-CVD group, *P* < 0.05; ^▲^Compared with MI group, *P* < 0.05; ^*$*^CVD compared with Non-CVD group.

CVD = MI + stroke + MS, the total number is 619.

The binary logistic regression results showed that age was a risk factor for CVDs in both genders. As age increased, the risk of developing CVDs increased exponentially, especially for female participants. Additionally, hypertension was an independent risk factor for MI or stroke in both genders; among those who had hypertension, the risk of suffering from MI or stroke was 5.26 times as much as those who had no hypertension among males, and this risk was approximately sixfold in females. Of the participants, the proportion of light-intensity PA (LPA) was only 10.2% (for males, the proportion was 14.3%; for females, the proportion was 7.2%), while 64.3% participated in VPA in their daily lives, and there were significant differences between males and females (*χ*^2^ = 251.05, *P* < 0.001). For MI or stroke patients, VPA was a protective factor in both genders; while MPA was a protective factor only in females.

### The associations of MI and stroke with PA in males and females of each age stratum

Tables 3 and 4 present the associations of MI, stroke, or MS with different levels of PA stratified by age. In the model, light-intensity PA was set as the reference group. The binary logistic regression analysis showed that after adjusting for age and gender, only VPA was positively associated with the incidence of stroke and MS in males, and with increasing age, the OR tended to increase (among participants < 45, the OR was 0.210, while among participants aged ≥ 75, the OR was 0.526). But the similar significant effect was not found among male MI patients. In females, the data showed that both moderate-intensity and vigorous-intensity PA can be beneficial for the prevention of MI and stroke patients aged more than 60 years.

**Table 3:** Effect of PA on MI or stroke among male participants stratified by age (OR (95%CI)).

	Age			
	< 45	45–59	60–74	75–
MI				
MPA	1.397 (0.126–15.460)	1.965 (0.759–5.088)	0.731 (0.360–1.486)	2.097 (0.678–6.493)
VPA	0.525 (0.048–5.804)	0.590 (0.234–1.492)	0.540 (0.284–1.026)	1.168 (0.363–3.758)
Stroke				
MPA	0.559 (0.149–2.094)	1.556 (0.869–2.786)	1.142 (0.773–1.688)	0.932 (0.515–1.689)
VPA*	0.210 (0.056–0.786)**	0.559 (0.323–0.969)**	0.631 (0.436–0.914)**	0.526 (0.283–0.979)**
MS				
MPA*	0.582 (0.177–1.920)	1.809 (1.075–3.043)**	0.240 (0.197–0.294)	1.029 (0.596–0.1.776)
VPA*	0.263 (0.084–0.818)**	0.602 (0.366–0.988)**	0.149 (0.120–0.164)**	0.578 (0.328–0.1.019)*

**Compared with light-level physical activity, *P* < 0.01; *indicates *P* < 0.05.

**Table 4:** Effect of PA on MI or stroke among **female** participants stratified by age (OR (95%CI)).

	Age			
	< 45	45–59	60–74	75–
MI				
MPA*	–	0.534 (0.140–2.041)	0.265 (0.134–0.527)**	0.856 (0.253–2.902)
VPA*	–	0.268 (0.077–0.933)**	0.257 (0.143–0.464)**	1.042 (0.333–3.262)
Stroke				
MPA*	–	1.669 (0.497–5.611)	0.467 (0.309–0.705)**	0.378 (0.182–0.783)**
VPA*	–	1.459 (0.455–4.675)	0.321 (0.219–0.471)**	0.415 (0.213–0.810)**
MS				
MPA*	–	1.102 (0.452–2.683)	0.154 (0.126–0.187)**	0.449 (0.234–0.862)**
VPA*	–	0.847 (0.365–1.966)	0.108 (0.095–0.123)**	0.545 (0.301–0.986)**

**Compared with light-level physical activity, *P* < 0.01; *indicates *P* < 0.05.

For people aged no more than 45 years, 1 was diagnosed with MI, 6 were diagnosed with MI or stroke.

### AUC of PA types and intensity for MI and stroke in males and females of each age stratum

To evaluate the prediction effect of different types of PA, a ROC analysis was conducted, and the results are shown in Tables 5 and 6. For male MI and stroke patients over 45 years of age and younger than 59, a sedentary lifestyle for a week had the largest AUC (0.715 for MI and 0.623 for stroke, *P* < 0.01), and the AUC of leisure sports for MI (0.611, *P* < 0.05) and stroke (0.612, *P* < 0.01) were higher than working or housework activity. In males over 60 years old, the AUC of a sedentary lifestyle for stroke was higher than that of any other physical activities (0.571 for the 60–74 age group, 0.621 for the 75– age group, *P* < 0.01), and similar results were found in female stroke patients over 60 (0.595 for the 60–74 age group, 0.640 for the 75– age group, *P* < 0.01). For female stroke patients younger than 45, the AUC of working or housework activity was the highest (0.658, *P* < 0.05).

**Table 5:** AUC of different PA types for MI and stroke stratified by age (AUC (95%CI)).

Age/PA type/disease	MI		Stroke	
	Male	Female	Male	Female
< 45				
Sedentary	0.431 (0.358–0.504)	0.402 (0.348–0.456)	0.432 (0.266–0.598)	0.374 (0.265–0.483)
Sleep	0.311 (0.190–0.511)	0.410 (0.269–0.552)	0.470 (0.285–0.654)	0.511 (0.186–0.836)
Working or housework	0.304 (0.073–0.535)	0.412 (0.323 (0.502))	0.234 (0.094–0.374)	0.658 (0.533–0.783)*
Leisure	0.555 (0.268–0.842)	0.303 (0.000–0.645)	0.354 (0.218–0.490)	0.430 (0.168–0.693)
Transportation	0.437 (0.151–0.722)	0.183 (0.000–0.391)	0.379 (0.214–0.544)	0.394 (0.115–0.673)
45–59				
Sedentary	0.715 (0.643–0.787) **	0.587 (0.462–0.712)	0.623 (1.572–1.675) **	0.545 (1.488–1.602)**
Sleep	0.422 (0.349–0.534)	0.527 (0.405–0.648)	0.566 (0.510–0.621)*	0.547 (0.489–0.606)
Working or housework	0.339 (0.261–0.417)**	0.385 (0.268–0.502)	0.282 (0.235–0.330)**	0.405 (0.355–0.455)**
Leisure	0.611 (0.510–0.711)*	0.518 (0.404–0.631)*	0.612 (0.552–0.673)**	0.510 (0.454–0.565)
Transportation	0.472 (0.381–0.563)	0.459 (0.358–0.560)	0.490 (0.433–0.548)	0.496 (0.442–0.551)
60–74				
Sedentary	0.535 (0.447–0.623)	0.560 (0.496–0.624)	0.571 (1.533–0.609)**	0.595 (1.562–1.629)**
Sleep	0.498 (0.420–0.576)	0.482 (0.414–0.550)	0.575 (0.539–0.612)**	0.556 (0.522–0.591)**
Working or housework	0.378 (0.305–0.452)**	0.413 (0.342–0.484)*	0.357 (0.324–0.390)**	0.369 (0.336–0.403)**
Leisure	0.541 (0.457–0.625)	0.496 (0.431–0.562)	0.534 (0.497–0.571)	0.515 (0.482–0.549)
Transportation	0.463 (0.385–0.540)	0.513 (0.447–0.579)	0.523 (0.486–0.560)	0.456 (0.423–0.489)*
75–				
Sedentary	0.466 (0.346–0.586)	0.503 (0.397–0.609)	0.621 (1.554–1.689)**	0.640 (1.565–1.715)**
Sleep	0.406 (0.294–0.518)	0.370 (0.247–0.493)*	0.503 (0.429–0.577)	0.558 (0.475–0.642)
Working or housework	0.482 (0.364–0.601)	0.449 (0.305–0.593)	0.396 (0.329–0.462)**	0.436 (0.355–0.517)
Leisure	0.542 (0.429–0.656)	0.559 (0.429–0.690)	0.497 (0.428–0.566)	0.462 (0.383–0.541)
Transportation	0.491 (0.377–0.604)	0.558 (0.438–0.679)	0.443 (0.377–0.509)	0.433 (0.354–0.511)

*P < 0.05;**P < 0.01.

**Table 6:** AUC of different PA intensity for MI and stroke stratified by age (AUC (95%CI)).

Age/PA intensity/disease	MI		Stroke	
	Male	Female	Male	Female
< 45				
MPA	0.577 (0.536–0.617)**	0.583 (0.535–0.630)**	0.622 (0.600–0.645)**	0.416 (0.391–0.440)
VPA	0.598 (0.558–0.637)**	0.536 (0.492–0.580)	0.584 (0.561–0.608)**	0.657 (0.490–0.823)
45–59				
MPA	0.577 (0.536–0.617)**	0.583 (0.535–0.630)**	0.622 (0.600–0.645)**	0.584 (0.560–0.609)**
VPA	0.598 (0.558–0.637)**	0.536 (0.492–0.580)	0.584 (0.561–0.607)**	0.540 (0.516–0.564)**
60–74				
MPA	0.577 (0.536–0.617)**	0.583 (0.535–0.630)**	0.622 (0.600–0.645)**	0.584 (0.560–0.609)**
VPA	0.598 (0.558–0.637)**	0.464 (0.420–0.508)	0.584 (0.561–0.607)**	0.540 (0.516–0.564)**
75–				
MPA	0.577 (0.536–0.617)**	0.417 (0.370–0.465)**	0.622 (0.600–0.645)**	0.584 (0.560–0.609)**
VPA	0.598 (0.558–0.637)**	0.536 (0.492–0.530)	0.584 (0.561–0.607)**	0.460 (0.436–0.484)

*P < 0.05;**P < 0.01.

We also calculated the total METs of moderate-intensity and vigorous-intensity PA for each age stratum for MI and stroke patients and then conducted the ROC analysis by gender (Table 6). We found an interesting result that there were no significant differences between the younger, mid-age, and older patients (*P* > 0.05). However, the AUC of VPA was higher than MPA for male MI patients, while the reverse results were found among female MI patients. For stroke patients, MPA had a higher AUC than VPA in both males and females.

### Estimating the PAR for multiple risk factors of stroke and MI among male and female patients

For MI, the incidence rate among males was slightly higher than that among females (1.56% VS 1.17%), and for stroke, the incidence rate among males was also higher than that among females (6.09% VS 4.48%). We calculated the *AF*_*exp*_ and population-attributable risk (PAR) of age for MI and stroke patients. The estimated attributable risk (AR) of gender among MI patients was 23.56%, and the PAR was 12.52%. For stroke patients, the *AF*_*exp*_ and PAR were 27.72% and 13.70, respectively; the male was set as the exposure factor. In addition, we also estimated the PAR of age, hypertension, and PA intensity among MI and stroke patients after adjusting for other risk factors. The results are stratified by gender and shown in Table 7.

**Table 7:** The attributable risk and population attributable risk for the five risk factors among MI and stroke patients (%).

Group/risk factors	Age		Hypertension		PA intensity		
	< 60	60–^#^	No	Yes^#^	LPA^**#**^	MPA	VPA
MI							
Males							
IR	0.86	2.87	1.32	2.73	1.83	2.43	1.19
RR		3.34		2.07	0.75/1.54	–	–
*AF*_*exp*_	–	70.07	–	51.66	–	− 32.57	35.09
*PAR*	–	*45.48*	–	*15.93*	–	− 10.57	9.12
Females							
IR	0.41	2.39	0.65	2.86	2.20	1.23	1.03
RR		5.83		4.4	1.79/2.14		
*AF*_*exp*_	–	82.97	–	77.15	–	44.17	53.05
*PAR*	–	*65.41*	–	*44.28*	–	*14.31*	*9.86*
Stroke							
Males							
IR	2.43	12.29	2.71	19.48	7.37	9.36	4.55
RR		5.58		7.19	0.78/1.62	–	–
*AF*_*exp*_	–	82.20	–	21.41	–	− 26.94	38.22
*PAR*	–	*61.52*	–	*17.24*	–	− *9.59*	*10.85*
Females							
IR	1.82	8.53	1.81	12.48	6.94	4.64	4.14
RR		4.69		6.89	1.50/1.67	–	–
*AF*_*exp*_	–	80.11	–	85.46	–	34.71	42.03
*PAR*	–	*60.42*	–	*60.56*	–	*9.89*	*6.42*

^#^Was calculated as an exposure risk factor because of the higher IR (incidence rate = diagnosed patients/total number of participants).

Significant values are in italics.

## Discussion

This large community-based cross-sectional study has revealed that the values of age, smoking, and drinking consistently tended to be increased among the participants with CVDs in both genders. It also demonstrated that the risk of MI or stroke increased with age, especially for people aged more than 75 years (OR = 33.169 and 53.292 for males and females, respectively). Moreover, the average age of MI or stroke patients was much higher than the age of the non-CVD participants. It has been confirmed that age itself or age-associated factors play a key role in MI and stroke. As reported by Oliveira et al.^40^, a significantly slower reperfusion rate and lower reperfusion magnitude were observed in older adults compared to young healthy adults, and the reperfusion rate in older adults was also slower than that in healthy older adults without CVD risk factors. In addition to the risk of cardiovascular disease events, the death risk is increased with each decade of age^41^ according to the Global Registry of Acute Coronary Events (GRACE) report^42^. Furthermore, older age contributed the highest PAR for MI and stroke in both genders and ranged from 45.48% to 65.41%. Hypertension ranged from 17.24% to 60.56% for female stroke patients, which indicated that females over 60 years old with hypertension were more likely to suffer a stroke. Therefore, we should not neglect the effects of aging per se on microvascular function and microvascular responsiveness impairment, especially for female cardiovascular disease patients. The results also indicated that educational background was a protective factor for cardiovascular disease in both genders, especially for females. For marital status, single people may have a higher risk of MI or stroke, and we also found that marriage was a negative risk factor in males after adjusting for age factors, which was mentioned by previous studies^43^. A systematic review^44^ evaluated the relationship between marital status and cardiovascular diseases and revealed that most studies showed better outcomes for married persons; in particular, single men generally had the poorest results. However, the review also revealed that being married was associated with obesity and thus may affect health status^44^. Moreover, we found significant differences in BMI, WC, VFI, and PBI (%) between the non-CVD participants and the participants with MI or stroke (*P* < 0.01). Notably, we also found that stroke patients had the highest BMI, WC, BM, VFI, and PBI (%).

It is clear that keeping exercise is a powerful medicine for the primary and secondary prevention of various chronic diseases^25^. In this study, the prevalence of VPA among participants without CVDs was 63.2% in males and 66.4% in females, while for patients with CVDs, the prevalence was no more than 50% in males and 60% among females. These results confirmed the protective effect of vigorous-intensity levels of PA on cardiovascular disease risk among CVDs patients in both genders after adjusting for age. However, moderate-level PA had no significant association with MI or stroke in male participants. When we combined MI and stroke as a single variable, MPA seemed to only have a protective effect on people aged younger than 60 years. According to the results of the logistic regression analysis, the OR of MPA was 1.319 when compared with low-level PA, and after we explored the age-stratified effect of PA on MI and stroke in males, there was no significant association between MI and moderate-level PA. Perhaps because the separate prevalence of stroke or MI was too small. Then, we analyzed the associations among female patients. For female cardiovascular disease patients, both moderate and vigorous levels of PA were protective factors, with ORs of 0.593 and 0.537, respectively. Those findings were not consistent with a previous study which was conducted in Shanghai, it suggested that regular participation in moderate-intensity PA was associated with reduced mortality, particularly CVD mortality^45^. We then analyzed the effect in different age subgroups, and significant protective effects were revealed for MI and stroke patients aged from 60 to 74 years. PA had no effect on MI in female patients aged more than 75 years, but a positive effect was found among patients aged from 45 to 59 years. Considering the small sample size of patients younger than 45, it is not enough to analyze the statistical effect. To further analyze the risk attribution of these factors, the results indicated that vigorous PA presented better long-term protective effects in both males and females, while moderate-intensity PA presented good long-term protective effects in females only. This finding was consistent with the latest research among UK populations^14^. Therefore, we can conclude that it is meaningful to estimate the associations between stroke, MI, and PA intensity among populations with different ages, and that exercise appears to be more protective in younger CVD patients.

Not only did the frequency of PA affect cardiovascular function, but PA type was associated with CVDs in our study as well. Previous studies on diabetes^46^, osteoporosis^47^, hypertension, coronary heart disease, and stroke^48^ have shown that different types of PA act differently. We analyzed the effect of sedentary time, sleep, working or housework, leisure activity, or transportation activity on MI or stroke. Among the patients aged 45–59 years old, the results showed that working or housework accounted for a significant protective effect on males with MI and stroke patients, especially on the females with stroke (the AUCs were 0.339, 0.282, and 0.405, respectively). But when compared with leisure time, working or housework showed a poorer protective effect, suggesting that younger patients with CVD could benefit more from leisure time activities. Similarly, working or housework activities were protective for MI patients older than 60 as well, but not for those who have a stroke in the same age group. Sedentary behavior was a noticeable risk factor among several kinds of chronic disease^49^, but no significant association between sedentary time and CVD risk was found for participant less than 45 years old in this study. However, the contribution of a sedentary lifestyle to MI or stroke was noticeable in most of the subgroups, and it was indicated as a risk factor in both genders, especially for the male patients in a middle-aged subgroup (45–59 years old). In addition, we found that sedentary behavior had a significant negative effect on stroke patients over the age of 60, regardless of their gender. Therefore, it seems that the correlation of sedentary behavior shows a stronger association with stroke than with MI.

## Conclusions and limitations

The current study indicated that age and hypertension were still the main risk factors for MI or stroke, but marriage status was a controversial factor that may be influenced by gender of participants. Vigorous-intensity PA had a better protective effect on cardiovascular health than moderate-level activity in both genders, while moderate-level PA could only affect patients younger than 60 years old. What’s more, female patients benefited more from moderate or high PA. Regarding the PA types, leisure time activity, working or housework activity were associated with fewer cardiovascular disease events, regardless males or females. And those protective effects were more significant among the younger. For the older patients aged over 60 years, the negative effect of sedentary behavior seemed more obvious. Finally, although we endeavored to control as many confounding factors as possible, we recruited a large population. Some limitations of this study still need to be considered. First, we only chose participants from Henan Province, which is not representative of the national population. Additionally, the cross-sectional design is limited by the lower power of the results, and it would be better to conduct retrospective or prospective studies. Finally, the IPAQ can only be used to assess habitual PA during the past 7 days, a variety of assessment tools may better reflect the relationship between PA and CVDs. Therefore, cohort studies with larger populations are needed in future studies, and the different effects of daily PA or habitual activity and aerobic exercise on CVDs should be further explored and analyzed.

## Data Availability

The datasets analyzed during the current study are not publicly available due to policy but are available from the corresponding author at reasonable request.

## References

[1] Li Y (2022). Global trends and regional differences in incidence and mortality of cardiovascular disease, 1990–2019: findings from 2019 global burden of disease study. Eur. J. Prev. Cardiol..

[2] WHO. *World Heart Day 2017*. [OL] 2017 [cited 2019 Sep.14th]; :[Available from: https://www.who.int/cardiovascular_diseases/world-heart-day-2017/en/.

[3] Tsao, C.W., et al., *Heart Disease and Stroke Statistics-2023 Update: A Report From the American Heart Association.* Circulation, 2023. 10.1161/CIR.0000000000001123 PMC12135016 36695182

[4] Zhao Q (2018). Changes in characteristics, risk factors, and in-hospital mortality among patients with acute myocardial infarction in the capital of China over 40 years. Int. J. Cardiol..

[5] Xi B (2014). The growing burden of cardiovascular diseases in China. Int. J. Cardiol..

[6] Aarnio K (2014). Long-term mortality after first-ever and recurrent stroke in young adults. Stroke.

[7] Ning X (2017). Increased stroke burdens among the low-income young and middle aged in rural China. Stroke.

[8] Zhao M (2017). Prevalence of cardiovascular medication on secondary prevention after myocardial infarction in China between 1995–2015: A systematic review and meta-analysis. PLoS ONE.

[9] Liu H (2017). Air pollution and hospitalization for acute myocardial infarction in China. Am. J. Cardiol..

[10] Lee IM (2012). Effect of physical inactivity on major non-communicable diseases worldwide: An analysis of burden of disease and life expectancy. Lancet.

[11] Lackland DT, Weber MA (2015). Global burden of cardiovascular disease and stroke: Hypertension at the core. Can. J. Cardiol..

[12] Dong K (2018). Stroke and (or) myocardial infarction attributable to modifiable risk factors in Henan, China. J. Am. Soc. Hypertens.

[13] Benjamin EJ (2019). Heart disease and stroke statistics-2019 update: A report from the American heart association. Circulation.

[14] Mu X (2022). Associations of physical activity intensity with incident cardiovascular diseases and mortality among 366,566 UK adults. Int. J. Behav. Nutr. Phys. Act.

[15] Holtermann A (2012). Occupational and leisure time physical activity: Risk of all-cause mortality and myocardial infarction in the Copenhagen City Heart Study. A prospective cohort study. BMJ Open.

[16] Krause N (2015). Occupational physical activity and 20-year incidence of acute myocardial infarction: Results from the Kuopio Ischemic Heart Disease Risk Factor Study. Scand. J. Work Environ. Health.

[17] Johnsen AM (2016). Association between occupational physical activity and myocardial infarction: A prospective cohort study. BMJ Open.

[18] Bennett DA (2017). Association of physical activity with risk of major cardiovascular diseases in Chinese men and women. JAMA Cardiol..

[19] Hidalgo-Santamaria M (2018). Physical activity intensity and cardiovascular disease prevention-from the Seguimiento Universidad de Navarra study. Am. J. Cardiol..

[20] Mury P (2017). Higher daily physical activity level is associated with lower RBC aggregation in carotid artery disease patients at high risk of stroke. Front. Physiol..

[21] Evenson KR (1999). Physical activity and ischemic stroke risk. The atherosclerosis risk in communities study. Stroke.

[22] Autenrieth CS (2013). Association between physical activity and risk of stroke subtypes: The atherosclerosis risk in communities study. Neuroepidemiology.

[23] Zhao S (2016). Association between time of day of sports-related physical activity and the onset of acute myocardial infarction in a chinese population. PLoS ONE.

[24] WHO, *WHO Guidelines Approved by the Guidelines Review Committee*, in *Global Recommendations on Physical Activity for Health*. 2010, World Health Organization Copyright (c) World Health Organization 2010.: Geneva.

[25] Bull FC (2020). World Health Organization 2020 guidelines on physical activity and sedentary behaviour. Br. J. Sports Med..

[26] Sofi F (2008). Physical activity during leisure time and primary prevention of coronary heart disease: An updated meta-analysis of cohort studies. Eur. J. Cardiovasc. Prev. Rehabil..

[27] Hu GC (2014). Occupational versus leisure-time physical activity in reducing cardiovascular risks and mortality among ethnic Chinese adults in Taiwan. Asia Pac. J. Public Health.

[28] Kubota Y (2017). Physical activity and lifetime risk of cardiovascular disease and cancer. Med. Sci. Sports Exerc..

[29] Holtermann A (2018). The physical activity paradox: Six reasons why occupational physical activity (OPA) does not confer the cardiovascular health benefits that leisure time physical activity does. Br. J. Sports Med..

[30] Allesoe K (2015). High occupational physical activity and risk of ischaemic heart disease in women: The interplay with physical activity during leisure time. Eur. J. Prev. Cardiol..

[31] Johansson MS (2022). The physical activity health paradox and risk factors for cardiovascular disease: A cross-sectional compositional data analysis in the Copenhagen City Heart Study. PLoS ONE.

[32] Jiang J (2016). Comparison of visceral and body fat indices and anthropometric measures in relation to untreated hypertension by age and gender among Chinese. Int. J. Cardiol..

[33] Yang Q (2018). Association of reproductive history with hypertension and prehypertension in Chinese postmenopausal women: A population-based cross-sectional study. Hypertens. Res..

[34] Wang Z (2014). Survey on prevalence of hypertension in China: background, aim, method and design. Int. J. Cardiol..

[35] Helou K (2017). Validity and reliability of an adapted arabic version of the long international physical activity questionnaire. BMC Public Health.

[36] Cleland C (2018). Validity of the International Physical Activity Questionnaire (IPAQ) for assessing moderate-to-vigorous physical activity and sedentary behaviour of older adults in the United Kingdom. BMC Med. Res. Methodol..

[37] Fan M, Lyu J, He P (2014). Chinese guidelines for data processing and analysis concerning the International Physical Activity Questionnaire. J. Zhonghua Epidemiol..

[38] Hu P (2018). Association between physical activity and abnormal glucose metabolism-A population-based cross-sectional study in China. J. Diabetes Complicat..

[39] Yu SZ (2007). Epidemiology and computer application-Third lecture rate difference and attribution ratio. Chin. J. Epidemiol..

[40] De Oliveira GV (2019). The effects of aging and cardiovascular risk factors on microvascular function assessed by near-infrared spectroscopy. Microvasc. Res..

[41] Castro-Dominguez Y, Dharmarajan K, McNamara RL (2018). Predicting death after acute myocardial infarction. Trends Cardiovasc. Med..

[42] Avezum A (2005). Impact of age on management and outcome of acute coronary syndrome: Observations from the Global Registry of Acute Coronary Events (GRACE). Am. Heart J..

[43] Otto CM (2018). Marital status and cardiovascular disease risk. Heart.

[44] Manfredini R (2017). Marital status, cardiovascular diseases, and cardiovascular risk factors: A review of the evidence. J. Womens Health (Larchmt).

[45] Liu Y (2018). Level of moderate-intensity leisure-time physical activity and reduced mortality in middle-aged and elderly Chinese. J. Epidemiol. Community Health.

[46] Kwon J (2019). Effects of different types and frequencies of physical activity on the homeostatic model assessment of insulin resistance. J. Investig. Med..

[47] Moreira LD (2014). Physical exercise and osteoporosis: Effects of different types of exercises on bone and physical function of postmenopausal women. Arq. Bras. Endocrinol. Metabol..

[48] Jia X (2018). Cardiovascular diseases in middle aged and older adults in China: The joint effects and mediation of different types of physical exercise and neighborhood greenness and walkability. Environ. Res..

[49] Tigbe WW (2017). Time spent in sedentary posture is associated with waist circumference and cardiovascular risk. Int. J. Obes. (Lond).

